# Lipopolysaccharide-Linked Enterobacterial Common Antigen (ECA_LPS_) Occurs in Rough Strains of *Escherichia coli* R1, R2, and R4

**DOI:** 10.3390/ijms21176038

**Published:** 2020-08-21

**Authors:** Anna Maciejewska, Marta Kaszowska, Wojciech Jachymek, Czeslaw Lugowski, Jolanta Lukasiewicz

**Affiliations:** Laboratory of Microbial Immunochemistry and Vaccines, Ludwik Hirszfeld Institute of Immunology and Experimental Therapy, Polish Academy of Sciences, Weigla 12, 53-114 Wroclaw, Poland; anna.maciejewska@hirszfeld.pl (A.M.); marta.kaszowska@hirszfeld.pl (M.K.); wojciech.jachymek@hirszfeld.pl (W.J.); czeslaw.lugowski@hirszfeld.pl (C.L.)

**Keywords:** enterobacterial common antigen, ECA, lipopolysaccharide, LPS, ECA_LPS_, endotoxin, NMR, mass spectrometry

## Abstract

Enterobacterial common antigen (ECA) is a conserved surface antigen characteristic for *Enterobacteriaceae*. It is consisting of trisaccharide repeating unit, →3)-α-d-Fuc*p*4NAc-(1→4)-β-d-Man*p*NAcA-(1→4)-α-d-Glc*p*NAc-(1→, where prevailing forms include ECA linked to phosphatidylglycerol (ECA_PG_) and cyclic ECA (ECA_CYC_). Lipopolysaccharide (LPS)-associated form (ECA_LPS_) has been proved to date only for rough *Shigella sonnei* phase II. Depending on the structure organization, ECA constitutes surface antigen (ECA_PG_ and ECA_LPS_) or maintains the outer membrane permeability barrier (ECA_CYC_). The existence of LPS was hypothesized in the 1960–80s on the basis of serological observations. Only a few *Escherichia coli* strains (i.e., R1, R2, R3, R4, and K-12) have led to the generation of anti-ECA antibodies upon immunization, excluding ECA_PG_ as an immunogen and conjecturing ECA_LPS_ as the only immunogenic form. Here, we presented a structural survey of ECA_LPS_ in *E. coli* R1, R2, R3, and R4 to correlate previous serological observations with the presence of ECA_LPS_. The low yields of ECA_LPS_ were identified in the R1, R2, and R4 strains, where ECA occupied outer core residues of LPS that used to be substituted by O-specific polysaccharide in the case of smooth LPS. Previously published observations and hypotheses regarding the immunogenicity and biosynthesis of ECA_LPS_ were discussed and correlated with presented herein structural data.

## 1. Introduction

Enterobacterial common antigen (ECA) is a common surface antigen present in Gram-negative bacteria belonging to the *Enterobacteriaceae* family [1]. The ECA, a heteropolysaccharide built of the trisaccharide repeating unit, →3)-α-d-Fuc*p*4NAc-(1→4)-β-d-Man*p*NAcA-(1→4)-α-d-Glc*p*NAc-(1→ [2], occurs as a cyclic form (ECA_CYC_), a phosphatidylglycerol (PG)-linked form (ECA_PG_), and lipopolysaccharide (LPS)-associated form (ECA_LPS_). LPS is the main surface antigen of Gram-negative bacteria that typically comprises of three structural components: lipid A, core oligosaccharide (OS), and the O-specific polysaccharide (O-PS; O-antigen determining O serotype) [3]. ECA_PG_ represents a major form of ECA and, together with LPS, is located on the cell surface, contributing to antigenicity, outer membrane integrity, and permeability, and finally, to viability and virulence of bacteria. Contrary to ECA_PG_ and ECA_LPS_ presented on the cell surface, ECA_CYC_ is located in the periplasm and has been recently pointed out as an important factor maintaining the outer membrane permeability barrier [4].

**Figure 1:**
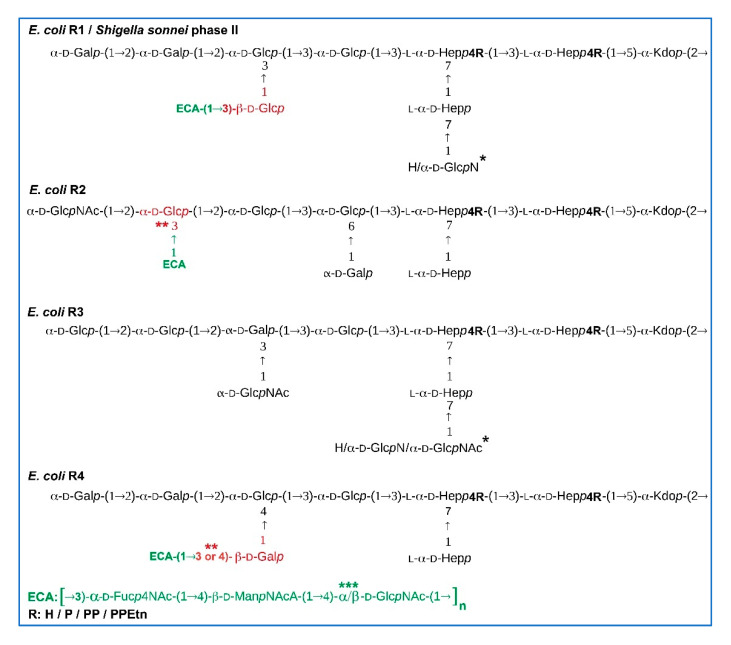
Structures of the core oligosaccharides (OS) and [ECA]-core OS in *S. sonnei* phase II and *E. coli* R1, R2, R3, and R4 lipooligosaccharides (LOS). The ECA stands for enterobacterial common antigen. The sugar residue of the ligation between core OS and ECA is colored in red. The ECA repeating unit is colored in green. R stands for nonstoichiometric substituents, such as a phosphate group (P), a pyrophosphate group (PP), pyrophosphorylethanolamine (PPEtn). The symbol * indicates heterogeneity of the core OS regarding terminal sugar residues. Symbol ** indicates ECA ligation sites assumed by the mass spectrometry only. Symbol *** indicates an inverted anomeric configuration of the D-Glc*p*NAc in the first ECA unit linked to the core OS, whereas an α-configuration is characteristic for the polymeric chain. The R1, R2, R3, and R4 core OS structures are presented according to published data [9,10,11].

Even though the existence and immunogenicity of ECA_LPS_ were suggested in the 1960s by Kunin et al. [5,6], it was relatively recent studies that demonstrated the first example of the covalent linkage between ECA and LPS in rough *Shigella sonnei* phase II [7,8]. Rough form of *S. sonnei* LPS, lipooligosaccharide (LOS) devoid of the O-PS, was partially substituted by ECA at the position occupied by the O-PS in the case of smooth *S. sonnei* phase I. Specifically, the distal β-d-Glc*p* residue in the outer core region of the chemotype R1 was substituted at position 3 by ECA [7,8] (Figure 1).

Kunin et al. predicted the existence of ECA_LPS_ by broad cross-reactivity of the anti-*E. coli* O14 rabbit serum with various rough and smooth *E. coli* strains. It was further demonstrated that only a few *E. coli* strains (serotype O14, O54, O124, and O144) elicited highly cross-reactive anti-ECA antibodies upon immunization of rabbits [6,12]. *E. coli* O14 was proven later on to be rough and synthesize LOS characterized by the R4 core chemotype [13,14]. Further studies demonstrated that ECA_LPS_-dependent immunogenicity generally was limited only to a few rough enterobacteria with complete core OS, such as, for example, *E. coli* R1, R4, and K-12 [15], *E. coli* R2 and R3 [15,16,17], *Proteus mirabilis* [18], *S. sonnei* phase II [19], and *Yersinia enterocolitica* O:3 and O:9 [20,21,22,23].

Nowadays, LPS (including O-PS) and ECA biosynthesis pathways are, to a great extent, well-described and share some similarities. Lipid A-core OS part is assembled on the cytoplasmic side of the inner membrane and translocated across the inner membrane. The O-PS is synthesized in a separate pathway, whereas both O-PS and ECA polysaccharides are produced via the Wzx/Wzy-dependent assembly pathway [3,24,25,26]. Completed O-PS is finally ligated to the lipid A-core OS by a WaaL ligase to form a mature LPS molecule ready for transport to the outer membrane [3]. Since repeating units of both polymers are assembled on the same lipid carrier—undecaprenyl pyrophosphate (Und-PP)—and undergone similar processing [25], key features of ECA_LPS_ structures are predicted partially on the basis of biosynthesis pathway analyses: (i) ECA_LPS_ can only be observed in strains incapable of producing the O-PS [3,25]; (ii) ECA is ligated to the core OS in the position used to be occupied by O-PS, and (iii) an inverted anomeric configuration of the D-Glc*p*NAc residue in the first ECA repeating unit linked to the core OS has to be observed, whereas an α-configuration is a characteristic for polymeric chain (Figure 1) [3]. All these presumptions were positively verified by our single case study on *S. sonnei* phase II ECA_LPS_ [7,8].

Successful identification of ECA_LPS_ in *S. sonnei* phase II prompted us to further survey for ECA_LPS_ in rough mutants of prototype *E. coli* R1, R2, R3, and R4 strains to correlate serological observations that were made before concerning the presence or absence of ECA_LPS_. *E. coli* O39 strain (PCM 209) characterized as the R1 core OS chemotype was selected for the purpose of seeking ECA_LPS_ presence in a smooth bacterium. ECA_LPS_ was identified in *E. coli* R1, R2, and R4, where it occupied the outer core region in the position that used to be substituted by O-PS in smooth LPS. No ECA_LPS_-derived fragments were found in *E. coli* R3. Several new examples of ECA_LPS_ presented herein were the preconditions for the further evaluation of biosynthesis and immunogenicity of this form of ECA. Finally, presented herein structural findings regarding ECA_LPS_ were discussed in relation to its origin (biosynthesis) and immunogenicity (serology).

## 2. Results

### 2.1. Isolation and Purification of E. coli R1, R2, R3, R4, and O39 ECA_LPS_-Derived Poly- and Oligosaccharides

**Figure 2:**
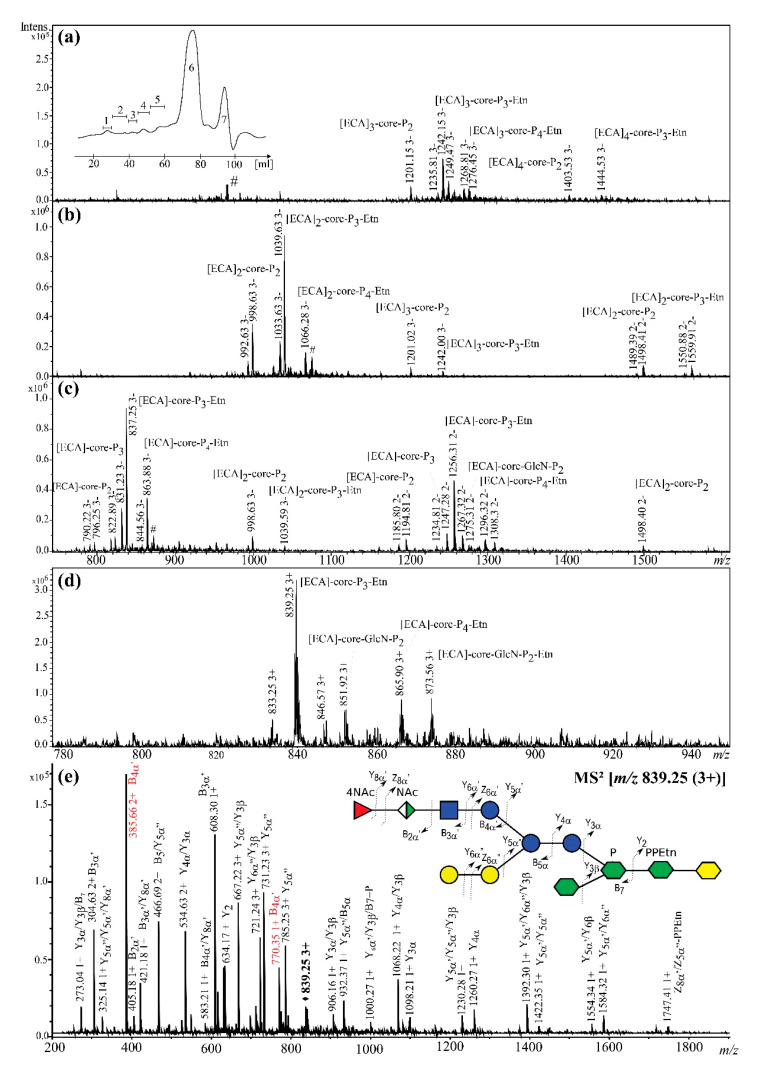
The electrospray ionization-ion trap (ESI) mass spectra of the [ECA]_n_-core OS identified in *E. coli* R1 LOS preparation obtained for (**a**) fraction 3 (negative-ion mode) with the Bio-Gel P-10 elution profile as the inset; (**b**) fraction 4 (negative-ion mode); (**c**) fraction 5 (negative-ion mode); (**d**) fraction 5 (positive-ion mode). (**e**) The positive-ion mode ESI-MS^2^ spectrum of the ion at *m/z* 839.25 (3+) attributed to [ECA]-core-P_3_-Etn glycoform, where core stands for Glc_3_-Gal_2_-Hep_3_-Kdo oligosaccharide. The symbol nomenclature for glycans was used for carbohydrates visual representation: 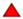 Fucose; 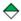 Mannuronic acid; 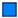 *N*-acetylglucosamine; 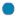 Glucose; 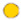 Galactose; 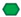 l,d-*manno*-Heptose; 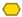 Kdo; NAc, *N*-acetyl group [27]. The interpretation of ions is shown in Table 1. The most informative ions are colored in red. The mark # stands for non-interpreted ions.

Poly- and oligosaccharides of ECA_LPS_ were searched for among products of *E. coli* LOS hydrolysis. Lipooligosaccharides (LOS) were extracted from bacterial cells by a hot phenol/water method. Purified LOS preparations were delipidated by mild acid hydrolysis and separated on Bio-Gel P-10. Alternative methods of poly- and oligosaccharide fractionation (gel chromatography; See Section 4.2) did not give better separation towards the identification of fragments of ECA_LPS_ than Bio-Gel P-10 (data not shown). For all selected strains, including *E. coli* PCM 209 strain, Bio-Gel P-10 elution profiles were similar with profiles previously described for *S. sonnei* phase II [7,8], yielding from one to seven pooled poly- and oligosaccharide fractions and suggesting a rough character of all LOS preparations, as was shown for *E. coli* R1 (Figure 2a, inset Bio-Gel P-10 profile).

Contrary to O39 serotype prediction for PCM 209 strain, the strain also appeared to be rough and was characterized by LPS core OS of the R1 chemotype. All fractions were analyzed by electrospray ionization-ion trap (ESI-IT) or matrix-assisted laser desorption/ionization-time of flight (MALDI-TOF) mass spectrometry (MS). The aim of MS analyses was the identification of core OS fractions substituted by at least one ECA repeating unit ([ECA]_n_-core). MS spectra interpretations were based on the previously published structures of ECA [2,28] and core OS of chemotypes R1 and R2 [9,10], R3 and R4 [10,11] (Figure 1). [ECA]_n_-core glycoforms were identified for *E. coli* R1, PCM 209 (R1 chemotype), R2, and R4, whereas no [ECA]-core OS was identified for *E. coli* R3, and that trait is discussed in detail in Section 2.2 and Section 2.3, respectively.

### 2.2. Rough Strains of E. coli R1, R2, and R4 Synthesize ECA_LPS_

*E. coli* R1 synthetized ECA_LPS_, as ESI-MS analyses of obtained Bio-Gel P-10 fractions showed ions attributed to [ECA]_3_-core OS glycoforms labeled in Figure 2 as [ECA]_n_-core-P_n_-Etn_n_, where the core stands for previously published sugar backbone of the R1 core OS [9,11]. Negative-ion mode ESI-MS profiles (data not shown) for fractions 1 and 2 did not match any ECA-derived polymers or ECA_CYC_, as was observed previously for *S. sonnei* phase II [7,8]. The fraction 3 consisted of [ECA]_3–4_-core OS glycoforms, constituting three to four ECA repeating units and characterized by heterogeneity regarding phosphate groups (P) and ethanolamine (Etn) substitution within the core region (Figure 2a, Table 1). For example, [ECA]_3_-core-P_2_, [ECA]_3_-core-P_3_-Etn, [ECA]_3_-core-P_4_-Etn, and [ECA]_4_-core-P_3_-Etn were identified.

The fraction 4 consisted of [ECA]_2-3_-core OS and was also defined by the heterogeneity of substitution with P and PEtn (Figure 2b, Table 1). Fraction 5 consisted mainly of [ECA]-core OS with a trace amount of [ECA]_2_-core. Two glycoforms were observed regarding the core OS region differing by the presence or the lack of the terminal GlcN residue in the inner core region (Figure 2c, Table 1, Figure 1). Interpretation of ions observed for fraction 6 was in agreement with the known structure of the free core OS of *E. coli* R1 (data not shown) [9,10]. Fraction 7 contained Kdo and Kdo-containing OS and degradation products of LOS hydrolysis (data not shown). The presence of the linkage between ECA and the core OS was further confirmed by a positive-ion mode ESI-MS^2^ analysis (Figure 2e). The ion at *m/z* 839.25 (3+), observed for fraction 5 and attributed to the [ECA]-Glc_3_-Gal_2_-Hep_3_-Kdo-P_3_-Etn glycoform (Figure 2e, inset structure; Table 1), was selected for ESI-MS^2^. The MS^2^ fragmentation showed a pattern of single, triple, and double-charged fragment ions of Y_i_, B_i_, and Z_i_ type, confirming the elucidated sequence of sugar residues, according to the nomenclature of Domon and Costello [29]. The profile of fragment ions matched the profile that was reported for the identical structure published previously for *S. sonnei* phase II [7,8]. Briefly, the ion at *m/z* 608.30 (1+) corresponded to singly protonated ECA trisaccharide (B3α’), whereas B4α’ ions at *m/z* 385.66 (2+) and *m/z* 770.35 (1+) matched the ECA repeating unit bound to an additional hexose, suggesting that the ECA chain was linked to terminal hexose of the outer core OS, what was additionally supported by other fragment ions.

**Figure 3:**
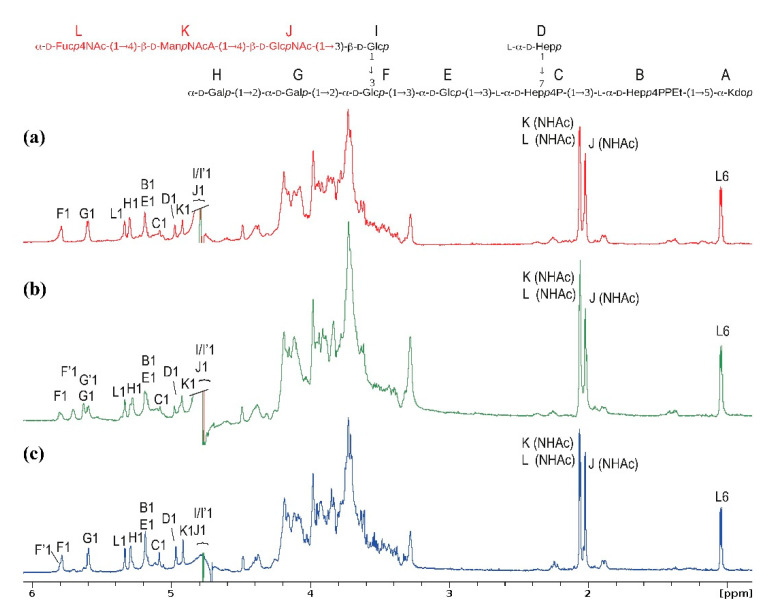
The 600 MHz ^1^H Nuclear Magnetic Resonance (NMR) spectra of the [ECA]-core OS glycoforms identified in fractions 5 of (**a**) *E. coli* R1; (**b**) *E. coli* PCM 209 (O39); (**c**) *S. sonnei* phase II LOS preparations [7,8]. The ECA repeating unit is colored in red. The capital letters refer to carbohydrate residues of the [ECA]-core OS, as shown in the inset structure. Letters with a prime sign denote residues of trace amounts of the core OS devoid of ECA. Chemical shift assignment for *E. coli* R1 is present in Table 2.

To identify the exact linkage position, fraction 5 was further investigated using one-dimensional (1D) (Figure 3a) and 2D ^1^H,^13^C,^31^P NMR spectroscopy (data not shown). The NMR spectra confirmed the structure of the [ECA]-core OS (Figure 3a, inset structure; Table 2), with characteristic ECA signals of three acetamide groups (residues K, L, J), the carboxylic group of →4)-β-d-Man*p*NAcA (residue K), and the methyl group of →3)-α-d-Fuc*p*4NAc (signal L6).

Moreover, interpreted NMR spectra (Table 2) indicated the identity of *E. coli* R1 [ECA]-core glycoform with the structure isolated from *S. sonnei* phase II characterized by R1 core chemotype (Figure 3c) [7,8]. Thus, the combined results also proved that ECA was linked to the LOS via the β(1→3) linkage between →4)-β-d-Glc*p*NAc-(1→ of ECA (residue J) and →3)-β-d-Glc*p* (residue I) of the outer core OS. Similarly to [ECA]-core OS of *S. sonnei* phase II, an inverted anomeric configuration of the D-Glc*p*NAc residue in the first ECA repeating unit linked to the core OS was observed, whereas an α-configuration was characteristic for the subsequent ECA repeating units (Figure 1 and Figure 3, inset structure). To track the presence of ECA_LPS_ in the strain *E. coli* PCM 209 serotyped as O39 and characterized by R1 core OS, we performed identical preparation and analytical protocol, as for rough strains. Contrary to O-serotype designation suggesting smooth morphology, the strain turned out to be rough. The elution profile of Bio-Gel P-10 separation (data not shown) was identical to *E. coli* R1 and *S. sonnei* phase II [7,8]. The fraction 5 was analyzed by ESI-MS^n^ (data not shown), where similar to *E. coli* R1 ion profile was observed with the ions attributed to [ECA]-core OS glycoforms. The presence of [ECA]-core in *E. coli* PCM 209 was further verified by 1D ^1^H (Figure 3b) and 2D NMR spectra (data not shown). The close similarity of both ECA-core glycoforms was demonstrated herein only by simply comparing the ^1^H NMR spectra of fractions 5 obtained for *E. coli* PCM 209 (R1 core chemotype), R1, and *S. sonnei* phase II (Figure 3a–c). 

**Figure 4:**
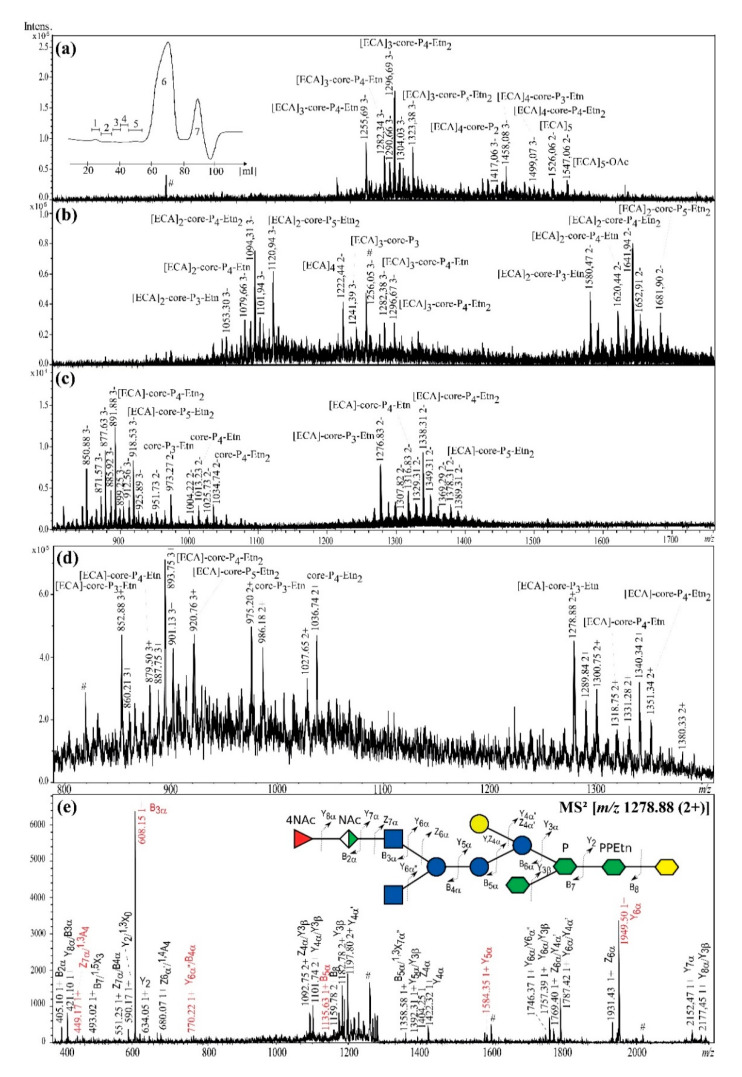
**The** ESI-MS^n^ mass spectra of [ECA]_n_-core OS identified in *E. coli* R2 LOS preparation: (**a**) in fraction 3 (negative-ion mode), inset the Bio-Gel P-10 elution profile; (**b**) in fraction 4 (negative-ion mode); (**c**) in fraction 5 (negative-ion mode); (**d**) in fraction 5 (positive-ion mode); (**e**) Positive-ion mode ESI-MS^2^ spectrum of the ion at *m/z* 1278.88 (+2) attributed to [ECA]-core-P_3_-Etn glycoform, where core stands for Glc_3_-GlcNAc-Gal-Hep_3_-Kdo oligosaccharide.. The interpretation of ions is shown in Table 3. The most informative ions are colored in red. The mark # stands for non-interpreted ions.

Furthermore, the identical analytical protocol was used to identify and analyze *E. coli* R2 [ECA]-core OS glycoforms. It was demonstrated that *E. coli* R2 also synthetized ECA_LPS_. The ESI-MS profiles for isolated fractions revealed the presence of various glycoforms of R2 core OS [9,10] substituted with ECA repeating units (Figure 4a–c; Table 3).

Negative-ion mode ESI-MS profiles for fraction 1 did not match any ECA-derived polymers (data not shown). Mass spectra of the fraction 2 contained triple-charged ions attributed to ECA-derived linear polymers built up of [ECA]_7_ and [ECA]_6_ and fragments thereof characterized by different O-acetylation pattern (data not shown). ESI-MS profiles of fractions 3, 4, and 5 were analogous to *E. coli* R1 and showed the presence of [ECA]_3–4_-core, [ECA]_2–3_-core, and [ECA]-core OS glycoforms, respectively (Figure 4a–c, Table 3). Additionally, linear [ECA]_5_ or [ECA]_4_ were identified in the fractions 3-4 and the unsubstituted core OS glycoforms in the fraction 5. Interpretation of ions observed for fraction 6 was in agreement with the known structure of the free core OS of *E. coli* R2 (data not shown) (Figure 1) [9,10].

The ion at *m/z* 1278.88 (2+) detected in positive-ion mode (Figure 4d) was used to confirm the linkage between ECA and core OS by ESI-MS^2^ (Figure 4e). The ion corresponded to the [ECA]-Glc_3_-GlcNAc-Gal-Hep_3_-Kdo-P_3_-Etn glycoform. The pattern of fragment ions (Y_i_, B_i_, Z_i_, X_i_, A_i_) not only supported previously published carbohydrate sequences for ECA and R2 core OS but also demonstrated the linkage between R2 core OS and one repeating unit of ECA (Figure 4e, inset structure).

Two high-intensity ions were identified as a result of the glycoform fragmentation into ECA trisaccharide [B3α’ at *m/z* 608.15 (1+)] and R2 core OS [Y6α at *m/z* 1949.50 (1+)]. The presence of ECA within the outer core region (the region of the terminal disaccharide α-d-Glc*p*NAc-(1→2)-α-d-Glc*p* present in Figure 1) was supported by B5α ion at *m/z* 1135.63 (1+) and Y5α at *m/z* 1584.35 (1+). Further in-depth fragment ions interpretation allowed to discriminate between the distal core OS GlcNAc and Glc as equally possible residues to be substituted by ECA repeating unit. The ion Y6α”/B4α at *m/z* 770.22 (1+) built up of ECA-Glc fragment pointed out specifically Glc as a residue substituted by ECA. This structure was additionally supported by the ion at *m/z* 449.17 (1+) attributed to the fragment ^1,3^A_4_/Z_7α_ of outer core Glc substituted by GlcNAc and ECA, which most probably indicated the position 3 of the →2)-α-d-Glc*p* as a place of substitution by ECA (Figure 1). Further analysis of the linkage between ECA and core OS by NMR spectroscopy was not possible due to the high heterogeneity of fraction 5 (Figure 4c,d) that resulted in the complexity of NMR spectra.

**Figure 5:**
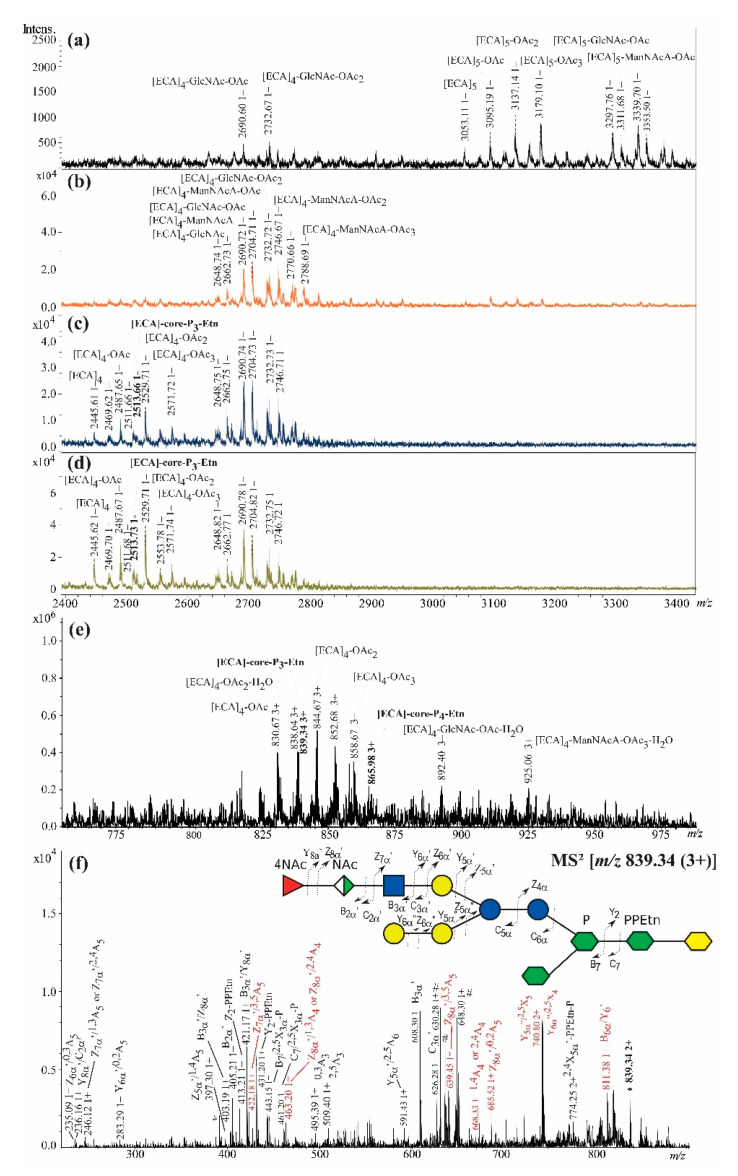
The mass spectra of ECA-containing fractions isolated from *E. coli* R4 LOS preparation. (**a**) The matrix-assisted laser desorption/ionization-time of flight (MALDI-TOF) negative-ion mode MS of the fraction 25; (**b**) the negative-ion mode MALDI-TOF MS of the fraction 26; (**c**) the negative-ion mode MS of the fraction 27; (**d**) the negative-ion mode MALDI-TOF MS of fraction 28; (**e**) the positive-ion mode ESI-MS spectrum of the fraction 28; (**f**) the ESI-MS^2^ spectrum of the ion at *m/z* 839.34 (3+) attributed to [ECA]-core-P_3_-Etn glycoform, where core stands for [ECA]-Gal_3_-Glc_2_-Hep_3_-Kdo oligosaccharide. The interpretation of ions is shown in Table 4. The most informative ions are colored in red. The mark # stands for non-interpreted ions.

For *E. coli* R4, the Bio-Gel P-10-based fractionation of poly- and oligosaccharides gave similar elution profile as for *E. coli* R1 and R2, which was characterized by 1–7 fractions (data not shown). Interpretation of the ions observed for fraction 6 was in agreement with previously published structures of the core OS of R4 chemotype (Figure 1) [10,11]. As *E. coli* R4 synthesized trace amounts of ECA_LPS_, fractions within the region corresponding to fraction 5 were not pooled, and every single fraction (collection tubes 25–28) was analyzed by MALDI-TOF MS. An interpretation of four representative fractions are presented in Figure 5a–d, showing a prevalence of the linear polymers [ECA]_4-5_ and their fragments over the low-intensity ions attributed to a few [ECA]-core OS glycoforms.

The fraction 25 was identified as linear polymers of ECA repeating units derived most probably from in-source fragmentation of ECA_PG_ and devoid of PG moiety and built up of [ECA]_4_ and [ECA]_5_ linear polymers and [ECA]_6_ and [ECA]_5_-derived degradation products with a different number of O-acetyl groups (OAc) (Figure 5a, Table 4). 

Fraction 26 was identified as linear [ECA]_4_ and [ECA]_5_-derived degradation products with different levels of OAc substitution (Figure 5b, Table 4). The fractions 27 and 28 had a similar composition to fraction 26 (Figure 5c,d, Table 4), but it contained low-intensity ions attributed to [ECA]-core OS, the ions at *m/z* 2513.73 (1−) and *m/z* 839.34 (3+) contributing to the [ECA]-Gal_3_-Glc_2_-Hep_3_-Kdo-P_3_-Etn, and the ion at *m/z* 865.98 (3+) contributing to the [ECA]-Gal_3_-Glc_2_-Hep_3_-Kdo-P_4_-Etn glycoform. The ion at *m/z* 839.34 (3+) detected in positive-ion mode (Figure 5e) was selected to confirm the linkage between ECA and core OS. The ion corresponded to the [ECA]-Gal_3_-Glc_2_-Hep_3_-Kdo-P_3_-Etn glycoform. The ESI-MS^2^ fragmentation pattern confirmed the linkage between ECA and core OS since a variety of fragments were identified containing both ECA and core OS residues (Figure 5f). Observed fragment ions of Y_i_, Z_i_, B_i_, C_i_, X_i_, and A_i_ (mostly single charged ions) supported the sugar sequence of the ECA and core OS region and were interpreted according to the nomenclature of Domon and Costello [29]. Variety of ions were identified as fragments of ECA part, i.e., the ions at *m/z* 236.16 (1+), 403.19 (1+), 405.21 (1+), 421.17 (1+), 495.31 (1+), 509.40, including the most abundant ion B_3_α’ [*m/z* 608.30 (1+)] and the C_3_α’ ion [*m/z* 626.28 (1+)], attributed to trisaccharide ECA repeating unit. Fragment ions representing the core OS region were also identified, for example, the ion at *m/z* 811.38 (1+) comprising five core OS hexoses (the Gal_3_-Glc_2_ fragment). The most informative for the identification of the ligation site were fragment ions comprising both ECA and core OS residues. The ions at *m/z* 463.20 (1+), 668.33 (1+), and 685.52 (1+) (comprising two or three residues of ECA and A_i_ fragment of the core OS hexose) pointed out for the terminal α-Gal*p* or β-Gal*p* as the ligation site for ECA, excluding →2)-α-Gal, →2,4)-α-Glc, and →3)-α-Glc (Figure 1). To discriminate between two terminal Gal residues as a place of ECA substitution, several single charged fragment ions were identified solely for one variant, where ECA was substituting β-Gal*p*-(1→4)-α-Glc*p* region of the core OS (*m/z* 422.18, 639.45, 685.52). It came to a similar conclusion by interpretation of the ion at *m/z* 740.80 (2+). Further, the ion Z7α’/^3,5^A_5_ at *m/z* 422.18 (1+) and the ion Z8α’/^3,5^A_5_ at *m/z* 639.45 (1+) indicated most probably the position 3 or 4 of terminal β-Gal as the linkage position of ECA (via GlcNAc residue of ECA) (Figure 1). Final differentiating between these positions was not possible on the basis of interpreted fragment ions. NMR analysis of the linkage was not possible due to the high heterogeneity of fraction 5 with a high prevalence of ECA-derived linear polymers (Figure 5d,e).

### 2.3. ECA_LPS_ is Absent in Rough E. coli R3 Lipooligosaccharide Preparation

**Figure 6:**
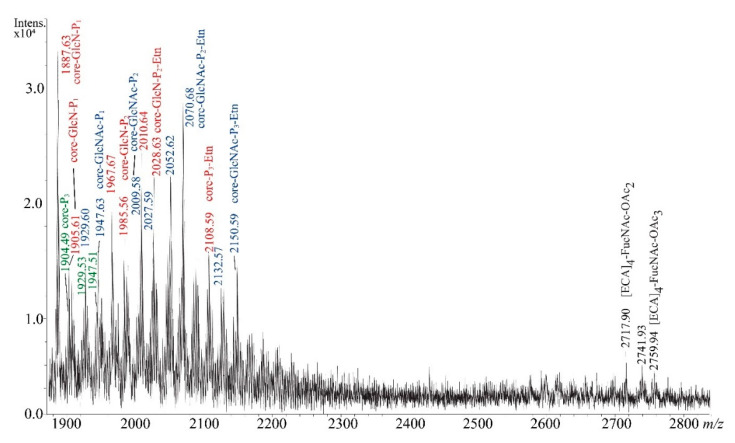
Negative-ion mode MALDI-TOF mass spectrum of fraction 5 isolated from *E. coli* R3 LOS preparation. The interpretation of ions is shown in Table 5. The core stands for Glc_3_-Gal-GlcNAc-Hep_3_-Kdo oligosaccharide. Colors green, red, and blue refer to three R3 core OS glycoforms (Figure 1).

The Bio-Gel P-10 elution profile of *E. coli* R3 poly- and oligosaccharides was similar to R1, R2, and R4 and also revealed the rough character of LOS R3 (1–7 fractions, data not shown). The MALDI-TOF mass spectrum of the fraction 5 did not show the presence of any ECA_LPS_-derived glycoforms (Figure 6).

Observed ions were limited to the core OS of R3 chemotype, according to previously published structures [10,11]. Three core OS glycoforms were identified and marked in colors in Figure 6 characterized by general schemes of the core-P_n_-Etn_n_, core-GlcN-P_n_-Etn_n_, and core-GlcN-P_n_-Etn_n_, where the core expression stands for Glc_3_-Gal-GlcNAc-Hep_3_-Kdo core OS backbone. The core-GlcN-P_n_-Etn_n_ glycoforms containing free GlcN were not reported before and were positively verified by NMR analysis of the fraction 5 (unpublished results). In the region of higher values of *m/z,* only trace amounts of ECA linear polymers were detected instead of searched core OS substituted with ECA (Table 5). No ECA_LPS_-derived polymers were identified in fractions 4, 3, 2, and 1.

## 3. Discussion

The story of ECA began with the discovery of ECA_LPS_ since the presence of ECA_LPS_ was originally inferred from serological cross-reactions in hemagglutination assays between patients’ sera and various *E. coli* O-serotypes during studies of urinary tract *E. coli* infections [5]. A few *E. coli* strains (serotypes O14, O54, O124, and O144) elicited highly cross-reactive antibodies in rabbits that could be removed from anti-O14 serum by absorption with extracts of any *E. coli* strain while retaining homological reactivity of the serum [6,12]. Described cross-reactivity was not related to O or K antigens. Thus, it was concluded that anti-O14 serum contained antibodies binding an antigen that had to be common for all *Enterobacteriaceae*, finally identified as ECA_PG_. Following this initial discovery of ECA, ECA_PG_ was identified, and its chemical structure and linkage to PG were elucidated [30]. Observed cross-reactivity suggested the presence of ECA as a non-immunogenic and an antigenic (ECA_PG_) and an immunogenic (ECA_LPS_) form. Ultimately, *E. coli* O14 proved to be rough strain expressing presumptive immunogenic ECA_LPS_ and characterized by the R4 chemotype of the core OS. Its roughness was masked by capsular antigen [31]. Finally, a presence of ECA_LPS_ in *E. coli* was suggested only for rough mutants expressing LOS with complete core OS, such as R1, R2, R4, and K-12 [15,17]. Conclusions described above were further supported by Whang et al. [16], who studied pairs of smooth parent strain and its corresponding rough mutant in case of *E. coli* R1 (strain F470), R2 (strain 576), R4 (O14), and R3 (strain F653), possessing complete core regions. Only rough and viable counterparts were able to elicit significant antibody response upon intravenous injection into rabbits. For heat-killed bacterial cells (100 °C, 1 h) used as an immunogen, only R1 and R4 (O14) were immunogenic.

Presented herein studies completed described serological and biochemical observation for *E. coli* with strong evidence supported by structural analysis. We utilized the protocol developed for *S. sonnei* phase II ECA_LPS_ to searching for this immunogenic form in the prototype *E. coli* R1, R2, R3, and R4. Analyzed strains were prototype strains that were used during pioneering studies on R1 (strain F470), R2 (strain F576), R3 (strain F653), and R4 (strain 2513) core OS structures [9,10,11]. The poly- and oligosaccharides comprising core OS substituted by ECA repeating units were identified in R1, R2, and R4. No ECA_LPS_-derived fragments were identified for *E. coli* R3 fractions. For rough *E. coli* R1 and PCM 209 (serotyped at the time of deposition as O39), the ESI-MS^2^ analysis supported by NMR analysis indicated that ECA occupied outer core residue that used to be substituted by O-PS in the case of smooth LPS. The same was only assumed with a high probability for R2 and R4 by MS since NMR analysis was impossible due to insufficient amounts of homogenous [ECA]-core samples (Figure 1). Structural results were in agreement with the previous hypothesis that the linking of ECA to the core OS of LPS was under genetic control [32,33]. The first indication of this phenomenon was based on the different behavior of two *E. coli* R1 mutants, F470 and F614, where the latter one was not immunogenic regarding ECA. Then, Schmidt et al. suggested the *E. coli* R1 probable connections between *rfaL* gene functionality and the expression of ECA immunogenicity in R4 and R1 strains. The *rfaL* gene (nowadays, referred to as WaaL in *E. coli*) encodes O-PS ligase responsible for glycosidic bond formation between O-PS and core OS of LPS [32,33].

Nowadays, LPS (O-PS) and ECA biosynthesis pathways are well described and share some similarities. The lipid A-core OS part is assembled on the cytoplasmic side of the inner membrane and translocated across the inner membrane. The O-PS part is synthesized in a separate pathway, whereas both O-PS and ECA polysaccharide are produced via the Wzx/Wzy-dependent assembly pathway [3,24,25,26]. Single repeating units of both polymers are assembled on the same lipid carrier—undecaprenyl pyrophosphate (Und-PP) [34]. For *E. coli*, the WecA enzyme is responsible for linkage formation between UndPP and sugar intermediate to promote subsequent repeating unit assembly. The same mechanisms of chain elongation are also used for both polymers [25]. Completed Und-PP-O-PS is finally ligated to lipid A-core OS by WaaL ligase to form a mature LPS molecule ready for transport to the outer membrane [3]. In the vast majority of bacterial species, the ECA present in the outer membrane is found covalently linked to the phosphatidylglycerol (ECA_PG_). ECA molecules bound to the lipid A can only be observed in bacterial strains incapable of producing the O-PS [1]. ECA_LPS_ occurrence used to be explained by key similarities between LPS and ECA biosynthesis described above, including Und-P carrier for precursor sugars, repeating units, and mature polymers [25,30].

A comparison of the biological repeating unit of the ECA and *E. coli* O-PS revealed one common feature for both polymers. Most of *E. coli* O antigens are synthesized via the Wzx/Wzy pathway, and d-GlcNAc serves as the initial sugar at the reducing end of O-PS that is attached to the core [35]. WecA-like transferases are specific for GlcNAc and initiate (by the formation of Und-PP-GlcNAc) the synthesis of ECA and most of *E. coli* O-PS [3]. In agreement with *S. sonnei* phase II ECA_LPS_ structure, D-GlcNAc of the first ECA repeating unit in the *E. coli* R1 and the PCM 209 was β-linked when it formed a bridge between the ECA and the core OS (Figure 1 and Figure 3; inset structure). The α configuration is present for subsequent ECA repeating units. Different anomeric configurations in the first and other repeating units in ECA is a result of the involvement of two different enzymes, polymerase Wzy (polymerization of the ECA chain) and ligase WaaL (O-PS and core ligation) [35].

It has been generalized that WaaL ligase can recognize a specific lipid A-core terminal structure to attach O-PS, and this has been established for *E. coli* R1, R2, and K-12 [3,36,37]. For R1 core OS, it is the outer core side branch β-Glc*p* [36]. This also applied to previously published *S. sonnei* phase II ECA_LPS_ [7,8]. The attachment site for O-PS in *E. coli* R2 is outer core α-Glc*p* residue [38], where additional terminal α-Glc*p*NAc is required for ligation activity [36]. Based on the high similarity between *E. coli* R1 and R4 WaaL proteins, it was assumed previously that the attachment site for the O-PS to R4 core OS is the side branch β-Gal*p* [36]. Herein, ECA ligation site to core OS-lipid A in selected rough *E. coli* R1, R2, and R4 strains fitted in the scheme of O-PS/core OS linkage. Moreover, ECA ligation to core OS matched also the overall assumption that the majority of Gram-negative bacteria could ligate other Und-PP-linked O-PS originated from various biosynthesis pathways [3]. This was previously demonstrated for *E. coli* K-12, where under specific conditions, a colanic acid could be covalently linked to lipid A-core by WaaL at the same attachment site position as for O-PS. Approximately 30% of core OS was substituted by colonic acid in *E. coli* K12 strain [39]. Herein, trace levels were detected for *E. coli* ECA_LPS_, especially in *E. coli* R4, suggesting WaaL specificity, acceptor/substrate interactions, and possibly other factors affecting ECA_LPS_ biosynthesis. Thus, ECA_LPS_ seemed to be a by-product of the reaction that attaches O-PS to LPS in the absence of O-PS substrates. This was demonstrated herein for *E. coli* PCM 209 (O39) strain with R1 core OS chemotype that appeared to switch from smooth to rough strain. Results and conclusions presented herein complemented ECA_LPS_ studies based on serological, biochemical, and genetic observation with structural data supporting previously published relationships between LPS, ECA, and ECA_LPS_ biosynthesis in *E. coli*.

## 4. Materials and Methods

### 4.1. Bacteria and Culture Conditions

Rough prototype strains of *E. coli* R1 (strain F470, a derivative of O8:K27-), R2 (strain F576, a derivative of O8:K42), R3 (F653; derivative of O111:K58), R4 (2513) were kindly donated by Professor Helmut Brade from the Research Center Borstel, Leibniz-Center for Medicine and Biosciences in Germany. The *E. coli* strain PCM 209 (O39) was obtained from the Polish Collection of Microorganisms (PCM) at the Ludwik Hirszfeld Institute of Immunology and Experimental Therapy, Polish Academy of Sciences (Wroclaw, Poland). Bacteria were grown in LB (5L flasks), as previously described [7,8]. After growing to logarithmic phase, bacteria were phenol-killed (final phenol concentration 0.5%), harvested by flow centrifugation (36,000 rpm; CEPA, Carl Padberg Zentrifugenbau GmbH, Lahr, Germany), and lyophilized.

### 4.2. Preparation of LOS and Oligosaccharides

Lipooligosaccharide fractions were extracted from bacterial cells using a hot phenol/water method and purified, as previously described [40]. Poly- and oligosaccharides were obtained by mild acid hydrolysis of LOS (1.5% acetic acid, 100 °C, 30 min), and the resulting mixtures were centrifuged (40,000 × *g*) using Beckman Coulter centrifuge (Beckman Coulter Life Sciences Division, Indianapolis, Indiana, USA), and the supernatant was collected, lyophilized, and fractionated on a Bio-Gel P-10 column (1.6 × 100 cm; Bio-Rad, Hercules, California, USA), as previously described [7,8]. Additionally, LOS-derived poly- and oligosaccharide preparations were fractionated TSK-gel G3000PW (*E. coli* O39, R3), TSK-gel G2500PW (*E. coli* O39) (Tosoh Corporation, Bioscience Division, Tokyo, Japan), Superdex 30 (*E. coli* R2) (GE Healthcare Bio-Sciences AB, Uppsala, Sweden) to test the efficiency of separation towards better purification of ECA_LPS_-derived glycoforms.

### 4.3. Electrospray Mass Spectrometry

ESI-MS and MS^2^ experiments were carried out on an amaZon SL ion trap (IT) mass spectrometer (Bruker Daltonik GmbH, Bremen, Germany) in both positive-ion and negative-ion modes. The oligosaccharide samples were dissolved in acetonitrile/water/formic acid solution (50:50:0.5; 50 g/mL). Source parameters were as follow: sample flow, 3 μL/min; ion source temperature, 200 °C; nitrogen flow, 5 L/min at a pressure of 8 psi. Spectra were scanned in the 200–2000 *m/z* range. The system was calibrated in positive-ion mode using the ESI-L tuning mix (Agilent Technologies, Santa Clara, California, USA) before acquisitions. MS^2^ experiments were performed using an isolation width of 4 *m/z*, an amplitude value started from 0.35, and a SmartFrag mode of 60–80%. Monoisotopic masses, *m/z* values, and fragment ions structures were calculated and interpreted using GlycoWorkbench software [41] using the nomenclature of Domon and Costello [29]. Symbol nomenclature for glycans (SNFG) was used for the visual representation of selected glycan structures [27].

### 4.4. Matrix-Assisted Laser Desorption/Ionization-Time of Flight Mass Spectrometry

Negative-ion mode MALDI-TOF MS of poly- and oligosaccharide fractions was carried out on a Bruker Ultraflextreme time-of-flight (TOF) instrument (Bruker Daltonik GmbH, Bremen, Germany). 2,5-Dihydroxybenzoic acid (10 mg/mL, acetonitrile/0.2 M citric acid, 1:1) was used as a matrix. External calibration in the negative-ion mode was applied using the Peptide Calibration Standard II (Bruker Daltonik, Bremen, Germany). Monoisotopic masses and *m/z* values were calculated and interpreted using GlycoWorkbench software [41].

### 4.5. NMR Spectroscopy

All NMR spectra were obtained using an Avance III 600 MHz (Bruker BioSpin GmbH, Rheinstetten, Germany) spectrometer equipped with a QCI-P cryoprobe (Bruker BioSpin GmbH, Rheinstetten, Germany). NMR spectra of isolated oligosaccharides were obtained in ^2^H_2_O (30 °C) using acetone as an internal reference (δ_H_ 2.225 ppm; δ_C_ 31.05 ppm). Oligosaccharides (1.8 mg) were repeatedly exchanged with ^2^H_2_O (99.95%) with intermediate lyophilization. The data were acquired and processed with standard Bruker software (TopSpin 3.1) (Bruker BioSpin GmbH, Rheinstetten, Germany) and assigned using SPARKY [42]. The signals were assigned based on two-dimensional experiments using correlation spectroscopy (COSY), clean total correlation spectroscopy (TOCSY), nuclear Overhauser effect spectroscopy (NOESY), heteronuclear multiple-bond correlation (HMBC), heteronuclear single quantum coherence-distortionless enhancement by polarization transfer (HSQC-DEPT), and HSQC-TOCSY. The mixing times in clean-TOCSY experiments were 30, 60, and 100 ms. The delay time in HMBC was 60 ms, and the mixing time for NOESY was 200 ms.

## Figures and Tables

**Table 1:** Interpretation of ESI-IT mass spectra of fractions 3–5 isolated from *E. coli* R1 LOS preparation.

Oligosaccharide Structure	Calculated Monoisotopic Mass (Da)	Observed Ion (m/z)	Calculated Ion (m/z)	Interpretation of the Ion
**Fraction 3 (negative mode)**				
[ECA]_3_-Glc_3_-Gal_2_-Hep_3_-Kdo-P_2_	3606.12	1201.15/nd	1201.03/1802.05	[M-3H]^3^^−^/[M-2H]^2^^−^
[ECA]_3_-Glc_3_-Gal_2_-Hep_3_-Kdo-P_3_-Etn	3729.13	1242.15/nd	1242.04/1863.56	[M-3H]^3^^−^/[M-2H]^2^^−^
[ECA]_3_-Glc_3_-Gal_2_-Hep_3_-Kdo-P_3_-Etn	3729.13	1235.81/nd	1236.03/1854.55	[M-H_2_O-3H]^3^^−^/[M-H_2_O-2H]^2^^−^
[ECA]_3_-Glc_3_-Gal_2_-Hep_3_-Kdo-P_3_-Etn	3729.13	1249.47/nd	1249.36/1874.54	[M-3H, Na]^3^^−^/[M-2H, Na]^2^^−^
[ECA]_3_-Glc_3_-Gal_2_-Hep_3_-Kdo-P_4_-Etn	3809.10	1268.81/nd	1268.69/1903.54	[M-3H]^3^^−^/[M-2H]^2^^−^
[ECA]_3_-Glc_3_-Gal_2_-Hep_3_-Kdo-P_4_-Etn	3809.10	1276.45/nd	1276.02/1914.53	[M-3H, Na]^3^^−^/[M-2H, Na]^2^^−^
[ECA]_4_-Glc_3_-Gal_2_-Hep_3_-Kdo-P_2_	4213.35	1403.53/nd	1403.44/2105.67	[M-3H]^3^^−^/[M-2H]^2^^−^
[ECA]_4_-Glc_3_-Gal_2_-Hep_3_-Kdo-P_3_-Etn	4336.35	1444.53/nd	1444.44/2167.17	[M-3H]^3^^−^/[M-2H]^2^^−^
**Fraction 4 (negative mode)**				
[ECA]_2_-Glc_3_-Gal_2_-Hep_3_-Kdo-P_2_	2998.90	998.63/1498.41	998.63/1498.44	[M-3H]^3^^−^/[M-2H]^2^^−^
[ECA]_2_-Glc_3_-Gal_2_-Hep_3_-Kdo-P_2_	2998.90	992.63/1489.39	992.62/1489.44	[M-H_2_O-3H]^3^^−^/[M-H_2_O-2H]^2^^−^
[ECA]_2_-Glc_3_-Gal_2_-Hep_3_-Kdo-P_3_-Etn	3121.91	1039.63/1559.91	1039.63/1559.95	[M-3H]^3^^−^/[M-2H]^2^^−^
[ECA]_2_-Glc_3_-Gal_2_-Hep_3_-Kdo-P_3_-Etn	3121.91	1033.63/1550.88	1033.62/1550.94	[M-H_2_O-3H]^3^^−^/[M-H_2_O-2H]^2^^−^
[ECA]_2_-Glc_3_-Gal_2_-Hep_3_-Kdo-P_4_-Etn	3201.88	1066.28/nd	1066.28/1599.93	[M-3H]^3^^−^/[M-2H]^2^^−^
[ECA]_3_-Glc_3_-Gal_2_-Hep_3_-Kdo-P_2_	3606.12	1201.02/nd	1201.03/1802.05	[M-3H]^3^^−^/[M-2H]^2−^
[ECA]_3_-Glc_3_-Gal_2_-Hep_3_-Kdo-P_3_-Etn	3729.13	1242.00/nd	1242.04/1863.56	[M-3H]^3-^/[M-2H]^2^^−^
**Fraction 5 (negative or positive mode)**				
[ECA]-Glc_3_-Gal_2_-Hep_3_-Kdo-P_2_	2391.68	796.25/1194.81	796.22/1194.83	[M-3H]^3^^−^/[M-2H]^2^^−^
[ECA]-Glc_3_-Gal_2_-Hep_3_-Kdo-P_2_	2391.68	790.22/1185.80	790.21/1185.82	[M-H_2_O-3H]^3^^−^/[M-H_2_O-2H]^2^^−^
[ECA]-Glc_3_-Gal_2_-Hep_3_-Kdo-P_3_	2471.64	822.89/1234.81	822.87/1234.82	[M-3H]^3^^−^/[M-2H]^2^^−^
[ECA]-Glc_3_-Gal_2_-Hep_3_-Kdo-P_3_-Etn	2514.69	837.25/1256.31	837.22/1256.34	[M-3H]^3^^−^/[M-2H]^2^^−^
[ECA]-Glc_3_-Gal_2_-Hep_3_-Kdo-P_3_-Etn	2514.69	831.23/1247.28	831.22/1247.33	[M-H_2_O-3H]^3^^−^/[M-H_2_O-2H]^2^^−^
[ECA]-Glc_3_-Gal_2_-Hep_3_-Kdo-P_3_-Etn	2514.69	844.56/1267.32	844.55/1267.32	[M-3H, Na]^3^^−^/[M-2H, Na]^2^^−^
[ECA]-Glc_3_-Gal_2_-Hep_3_-Kdo-GlcN-P_2_	2552.75	nd/1275.31	849.91/1275.37	[M-3H]^3^^−^/[M-2H]^2^^−^
[ECA]-Glc_3_-Gal_2_-Hep_3_-Kdo-P_4_-Etn	2594.65	863.88/1296.32	863.88/1296.32	[M-3H]^3^^−^/[M-2H]^2^^−^
[ECA]_2_-Glc_3_-Gal_2_-Hep_3_-Kdo-P_2_	2998.90	998.63/1498.40	998.63/1498.44	[M-3H]^3^^−^/[M-2H]^2^^−^
[ECA]_2_-Glc_3_-Gal_2_-Hep_3_-Kdo-P_3_-Etn	3121.91	1039.59/nd	1039.63/1559.95	[M-3H]^3^^−^/[M-2H]^2^^−^
[ECA]-Glc_3_-Gal_2_-Hep_3_-Kdo-P_3_-Etn	2514.69	839.25/1258.34 *	839.24/1258.35	[M+3H]^3+^/[M+2H]^2+^
[ECA]-Glc_3_-Gal_2_-Hep_3_-Kdo-P_3_-Etn	2514.69	833.25/nd	833.23/1249.34	[M-H_2_O+3H]^3+^/[M-H_2_O+2H]^2+^
[ECA]-Glc_3_-Gal_2_-Hep_3_-Kdo-P_3_-Etn	2514.69	846.57/1269.34 *	846.56/1269.34	[M+3H, Na]^3+^/[M+2H, Na]^2+^
[ECA]-Glc_3_-Gal_2_-Hep_3_-Kdo-GlcN-P_2_	2552.75	851.92/1277.31 *	851.92/1277.38	[M+3H]^3+^/[M+2H]^2+^
[ECA]-Glc_3_-Gal_2_-Hep_3_-Kdo-GlcN-P_2_-Etn	2617.76	873.56/nd	873.59/1309.89	[M+3H, Na]^3+^/[M+2H, Na]^2+^
[ECA]-Glc_3_-Gal_2_-Hep_3_-Kdo-P_4_-Etn	2594.65	865.90/1298.32 *	865.89/1298.33	[M+3H]^3+^/[M+2H]^2+^

ESI-IT, electrospray ionization-ion trap; LOS, lipoligosaccharide; [ECA], trisaccharide biological repeating unit of linear enterobacterial common antigen (ECA): →3)-α-d-Fuc*p*4NAc-(1→4)-β-d-Man*p*NAcA-(1→4)-α-d-Glc*p*NAc-(1→, where →4)-β-d-Glc*p*NAc-(1→ is present in the first repeating unit substituting core oligosaccharide (OS); Glc, glucose; Gal, galactose; Hep, heptose; Kdo, 3-deoxy-d-*manno*-octulosonic acid; GlcN, glucosamine; P, phosphate group; Etn, ethanolamine. nd, non-detected; * the MS region of double-charged ions is not shown in Figure 2d,e.

**Table 2:** The ^1^H and ^13^C NMR chemical shifts and selected inter-residue correlations from NOESY and HMBC spectra of [ECA]-core glycoform isolated from *E. coli* R1.

Residue		Chemical Shifts (ppm)								Selected Inter-Residue NOE and ^3^*J*_H,C_ Connectivities	
		H1 C1	H2,(H3′) C2	H3 C3	H4 C4	H5 C5	H6,H6′ C6	H7, H7′ C7	H8,H8′ C8	H1/C1 Connectivities to	Inter-Residue Atom/Residue
**A**	→5)-α-Kdo*p*	nd	(2.27) 96.2	1.91 34.2	4.13 66.4	4.18 73.4	3.70 69.9	3.81 73.0	3.47, 3.94 64.9		
**B**	→3)-l-α-d-Hep*p*4PPEtn-(1→	5.21 100.2	4.02 71.8	4.09 78.6	4.64 72.4	4.24 72.1	4.11 69.4	3.73 63.9		4.18	H5 of A
**C**	→3,7)-l-α-d-Hep*p*4P-(1→	5.11 103.7	4.40 70.6	4.12 79.9	4.42 69.5	3.81 73.3	4.23 68.8	3.61, 3.77 68.5		4.09 78.6	C3, H3 of B
**D**	l-α-d-Hep*p*-(1→	5.00 100.3	3.94 70.9	3.89 71.5	3.86 67.0	3.63 72.0	4.05 69.6	3.64, 3.75 63.7		3.61, 3.77 ^a^ 68.5	C7, H7,7′ of C
**E**	→3)-α-d-Glc*p*-(1→	5.21 102.1	3.68 71.2	4.09 76.8	3.80 71.3	3.92 73.2	3.81, 3.92 60.7			4.12 ^a^	H3 of C
**F**	→2,3)-α-d-Glc*p*-(1→	5.81 95.4	3.88 73.5	4.18 78.7	3.58 68.8	4.11 72.1	3.79, 4.00 61.1			4.09 76.8	C3, H3 of E
**G**	→2)-α-d-Gal*p*-(1→	5.63 92.1	4.00 73.2	4.20 68.9	3.99 70.9	4.15 72.2	3.76 62.1			3.88 ^a^	H2 of F
**H**	α-d-Gal*p*-(1→	5.32 96.6	3.86 69.2	3.96 70.2	4.00 70.2	4.14 72.1	3.75 62.1			4.00	H2 of G
**I**	→3)-β-d-Glc*p*-(1→	4.74 103.2	3.39 73.7	3.70 85.5	3.50 69.0	3.45 76.3	3.75, 3.91 61.5			4.18 78.7	C3, H3 of F
**I’ ^b^**	β-d-Glc*p*-(1→	4.76 103.2	3.34 74.0	3.53 76.7	3.43 70.5	3.45 76.6	3.92, 3.74 61.5			4.18 78.7	C3, H3 of F
**J**	→4)-β-d-Glc*p*NAc-(1→	4.79 102.4	3.76 56.4	3.75 72.8	3.70 79.6	3.55 75.2	3.73, 3.86 61.0			3.70 85.5	C3, H3 of I
**K**	→4)-β-d-Man*p*NAcA-(1→	4.94 99.8	4.51 54.3	4.08 73.3	3.84 74.9	3.88 77.2	- 175.1			3.70 ^a^ 79.6	C4, H4 of J
**L**	α-d-Fuc*p*4NAc-(1→	5.36 99.7	3.65 69.3	3.97 69.2	4.21 54.7	4.19 66.7	1.06 16.3			3.84	H4 of K
	PPEtn	4.21 63.2	3.30 40.8								

Anomeric configuration was determined on the basis of ^3^*J*_H1,C1_ coupling constant for following residues: B—174 Hz; C—171 Hz; D—172 Hz; E—173 Hz; F—175 Hz; G—172 Hz; H—173 Hz; I—163 Hz; J—162 Hz; K—163 Hz; L—175 Hz. ^a^ The value represents NOE connectivity only; ^b^ Residue I’ is a terminal residue I present in the core OS that was devoid of ECA trisaccharide; nd—not determined.

**Table 3:** Interpretation of ESI-IT mass spectra of [ECA]_2–4_-core OS glycoforms (fractions 3–5) isolated from *E. coli* R2 LOS preparation.

Oligosaccharide Structure	Calculated Monoisotopic Mass (Da)	Observed Ion (*m/z*)	Calculated Ion (*m/z*)	Interpretation of the Ion
**Fraction 3 (negative mode)**				
[ECA]_5_	3054.12	nd/1526.06	1017.03/1526.05	[M-3H]^3^^−^/[M-2H]^2^^−^
[ECA]_5_-OAc	3096.13	nd/1547.06	1031.04/1547.06	[M-3H]^3^^−^/[M-2H]^2^^−^
[ECA]_3_-Glc_3_-GlcNAc-Gal-Hep_3_-Kdo-P_3_-Etn	3770.16	1255.69/nd	1255.71/1884.07	[M-3H]^3^^−^/[M-2H]^2^^−^
[ECA]_3_-Glc_3_-GlcNAc-Gal-Hep_3_-Kdo-P_4_-Etn	3850.12	1282.34/nd	1282.37/1924.06	[M-3H]^3^^−^/[M-2H]^2^^−^
[ECA]_3_-Glc_3_-GlcNAc-Gal-Hep_3_-Kdo-P_4_-Etn_2_	3893.17	1290.66/nd	1290.71/1936.57	[M-H_2_O-3H]^3^^−^/[M-H_2_O-2H]^2^^−^
[ECA]_3_-Glc_3_-GlcNAc-Gal-Hep_3_-Kdo-P_4_-Etn_2_	3893.17	1296.69/nd	1296.72/1945.58	[M-3H]^3^^−^/[M-2H]^2^^−^
[ECA]_3_-Glc_3_-GlcNAc-Gal-Hep_3_-Kdo-P_4_-Etn_2_	3893.17	1304.03/nd	1304.04/1956.56	[M-3H, Na]^3^^−^/[M-2H, Na]^2^^−^
[ECA]_3_-Glc_3_-GlcNAc-Gal-Hep_3_-Kdo-P_5_-Etn_2_	3973.13	1323.38/nd	1323.37/1985.56	[M-3H]^3^^−^/[M-2H]^2^^−^
[ECA]_4_-Glc_3_-GlcNAc-Gal-Hep_3_-Kdo-P_2_	4254.37	1417.06/nd	1417.12/2126.18	[M-3H]^3^^−^/[M-2H]^2^^−^
[ECA]_4_-Glc_3_-GlcNAc-Gal-Hep_3_-Kdo-P_3_-Etn	4377.38	1458.08/nd	1458.12/2187.68	[M-3H]^3^^−^/[M-2H]^2^^−^
[ECA]_4_-Glc_3_-GlcNAc-Gal-Hep_3_-Kdo-P_4_-Etn_2_	4500.39	1499.07/nd	1499.12/2249.19	[M-3H]^3^^−^/[M-2H]^2^^−^
**Fraction 4 (negative mode)**				
[ECA]_4_	2446.90	nd/1222.44	814.63/1222.44	[M-3H]^3^^−^/[M-2H]^2^^−^
[ECA]_2_-Glc_3_-GlcNAc-Gal-Hep_3_-Kdo-P_3_-Etn	3162.94	1053.30/1580.47	1053.30/1580.46	[M-3H]^3^^−^/[M-2H]^2^^−^
[ECA]_2_-Glc_3_-GlcNAc-Gal-Hep_3_-Kdo-P_4_-Etn	3242.90	1079.66/1620.44	1079.96/1620.44	[M-3H]^3^^−^/[M-2H]^2^^−^
[ECA]_2_-Glc_3_-GlcNAc-Gal-Hep_3_-Kdo-P_4_-Etn_2_	3285.94	1094.31/1641.94	1094.31/1641.96	[M-3H]^3^^−^/[M-2H]^2^^−^
[ECA]_2_-Glc_3_-GlcNAc-Gal-Hep_3_-Kdo-P_4_-Etn_2_	3285.94	1101.94/1652.91	1101.63/1652.95	[M-3H, Na]^3^^−^/[M-2H, Na]^2^^−^
[ECA]_2_-Glc_3_-GlcNAc-Gal-Hep_3_-Kdo-P_5_-Etn_2_	3365.91	1120.94/1681.90	1120.96/1681.95	[M-3H]^3^^−^/[M-2H]^2^^−^
[ECA]_3_-Glc_3_-GlcNAc-Gal-Hep_3_-Kdo-P_3_	3727.12	1241.39/nd	1241.36/1862.55	[M-3H]^3^^−^/[M-2H]^2^^−^
[ECA]_3_-Glc_3_-GlcNAc-Gal-Hep_3_-Kdo-P_3_-Etn	3770.16	1255.03/nd	1255.71/1884.07	[M-3H]^3^^−^/[M-2H]^2^^−^
[ECA]_3_-Glc_3_-GlcNAc-Gal-Hep_3_-Kdo-P_4_-Etn	3850.12	1282.38/nd	1282.37/1924.06	[M-3H]^3^^−^/[M-2H]^2^^−^
[ECA]_3_-Glc_3_-GlcNAc-Gal-Hep_3_-Kdo-P_4_-Etn_2_	3893.17	1296.67/nd	1296.72/1945.58	[M-3H]^3^^−^/[M-2H]^2^^−^
**Fraction 5 (negative/positive modes)**				
Glc_3_-GlcNAc-Gal-Hep_3_-Kdo-P_3_	1905.45	nd/951.73	634.14/951.72	[M-3H]^3^^−^/[M-2H]^2^^−^
Glc_3_-GlcNAc-Gal-Hep_3_-Kdo-P_3_-Etn	1948.49	nd/973.27	648.49/973.24	[M-3H]^3^^−^/[M-2H]^2^^−^
Glc_3_-GlcNAc-Gal-Hep_3_-Kdo-P_4_-Etn	2028.46	nd/1004.22	669.14/1004.21	[M-H_2_O-3H]^3^^−^/[M-H_2_O-2H]^2^^−^
Glc_3_-GlcNAc-Gal-Hep_3_-Kdo-P_4_-Etn	2028.46	nd/1013.23	675.15/1013.22	[M-3H]^3^^−^/[M-2H]^2^^−^
Glc_3_-GlcNAc-Gal-Hep_3_-Kdo-P_4_-Etn_2_	2071.50	nd/1025.73	683.49/1025.73	[M-H_2_O-3H]^3^^−^/[M-H_2_O-2H]^2^^−^
Glc_3_-GlcNAc-Gal-Hep_3_-Kdo-P_4_-Etn_2_	2071.50	nd/1034.74	689.49/1034.74	[M-3H]^3^^−^/[M-2H]^2^^−^
[ECA]-Glc_3_-GlcNAc-Gal-Hep_3_-Kdo-P_3_-Etn	2555.71	850.88/1276.83	850.90/1276.85	[M-3H]^3^^−^/[M-2H]^2^^−^
[ECA]-Glc_3_-GlcNAc-Gal-Hep_3_-Kdo-P_4_-Etn	2635.68	871.57/1307.82	871.55/1307.82	[M-H_2_O-3H]^3^^−^/[M-H_2_O-2H]^2^^−^
[ECA]-Glc_3_-GlcNAc-Gal-Hep_3_-Kdo-P_4_-Etn	2635.68	877.63/1316.83	877.55/1316.83	[M-3H]^3^^−^/[M-2H]^2^^−^
[ECA]-Glc_3_-GlcNAc-Gal-Hep_3_-Kdo-P_4_-Etn_2_	2678.72	885.92/1329.31	885.89/1329.35	[M-H_2_O-3H]^3-^/[M-H_2_O-2H]^2^^−^
[ECA]-Glc_3_-GlcNAc-Gal-Hep_3_-Kdo-P_4_-Etn_2_	2678.72	891.88/1338.31	891.90/1338.35	[M-3H]^3^^−^/[M-2H]^2^^−^
[ECA]-Glc_3_-GlcNAc-Gal-Hep_3_-Kdo-P_4_-Etn_2_	2678.72	899.25/1349.31	899.22/1349.34	[M−3H, Na]^3^^−^/[M−2H, Na]^2^^−^
[ECA]-Glc_3_-GlcNAc-Gal-Hep_3_-Kdo-P_5_-Etn_2_	2758.69	912.56/1369.29	912.55/1369.33	[M-H_2_O-3H]^3^^−^/[M-H_2_O-2H]^2^^−^
[ECA]-Glc_3_-GlcNAc-Gal-Hep_3_-Kdo-P_5_-Etn_2_	2758.69	918.53/1378.31	918.56/1378.34	[M-3H]^3^^−^/[M-2H]^2^^−^
[ECA]-Glc_3_-GlcNAc-Gal-Hep_3_-Kdo-P_5_-Etn_2_	2758.69	925.89/1389.31	925.88/1389.32	[M-3H, Na]^3^^−^/[M-2H, Na]^2^^−^
Glc_3_-GlcNAc-Gal-Hep_3_-Kdo-P_3_-Etn	1948.49	nd/975.20	650.50/975.25	[M+3H]^3+^/[M+2H]^2+^
Glc_3_-GlcNAc-Gal-Hep_3_-Kdo-P_3_-Etn	1948.49	nd/986.18	657.83/986.24	[M+3H, Na]^3+^/[M+2H, Na]^2+^
Glc_3_-GlcNAc-Gal-Hep_3_-Kdo-P_4_-Etn_2_	2071.50	nd/1027.65	685.50/1027.75	[M-H_2_O+3H]^3+^/[M-H_2_O+2H]^2+^
Glc_3_-GlcNAc-Gal-Hep_3_-Kdo-P_4_-Etn_2_	2071.50	nd/1036.74	691.51/1036.76	[M+3H]^3+^/[M+2H]^2+^
[ECA]-Glc_3_-GlcNAc-Gal-Hep_3_-Kdo-P_3_-Etn	2555.71	852.88/1278.88	852.91/1278.86	[M+3H]^3+^/[M+2H]^2+^
[ECA]-Glc_3_-GlcNAc-Gal-Hep_3_-Kdo-P_3_-Etn	2555.71	860.21/1289.84	860.24/1289.85	[M+3H, Na]^3+^/[M+2H, Na]^2+^
[ECA]-Glc_3_-GlcNAc-Gal-Hep_3_-Kdo-P_3_-Etn	2555.71	nd/1300.75	867.57/1300.85	[M+3H, 2Na]^3+^/[M+2H, 2Na]^2+^
[ECA]-Glc_3_-GlcNAc-Gal-Hep_3_-Kdo-P_4_-Etn	2635.68	879.50/1318.75	879.57/1318.85	[M+3H]^3+^/[M+2H]^2+^
[ECA]-Glc_3_-GlcNAc-Gal-Hep_3_-Kdo-P_4_-Etn_2_	2678.72	887.75/1331.28	887.91/1331.36	[M-H_2_O+3H]^3+^/[M-H_2_O+2H]^2+^
[ECA]-Glc_3_-GlcNAc-Gal-Hep_3_-Kdo-P_4_-Etn_2_	2678.72	893.75/1340.34	893.91/1340.37	[M+3H]^3+^/[M+2H]^2+^
[ECA]-Glc_3_-GlcNAc-Gal-Hep_3_-Kdo-P_4_-Etn_2_	2678.72	901.13/1351.34	901.24/1351.35	[M+3H, Na]^3+^/[M+2H, Na]^2+^
[ECA]-Glc_3_-GlcNAc-Gal-Hep_3_-Kdo-P_5_-Etn_2_	2758.69	920.76/1380.33	920.57/1380.35	[M+3H]^3+^/[M+2H]^2+^

OAc—O-acetyl group.

**Table 4:** Interpretation of matrix-assisted laser desorption/ionization-time of flight (MALDI-TOF) mass spectra of ECA-containing fractions 25, 26, 27, and 28 isolated from *E. coli* R4 LOS preparation.

Oligosaccharide Structure	Calculated Monoisotopic Mass (Da)	Observed Ion (*m/z*)	Calculated Ion (*m/z*)	Interpretation of the Ion
**Fraction 25 (negative mode)**				
[ECA]_5_-OAc	3096.13	3095.19	3095.13	[M-H]^−^
[ECA]_5_	3054.12	3053.11	3053.12	[M-H]^−^
[ECA]_5_-OAc_2_	3138.14	3137.14	3137.14	[M-H]^−^
[ECA]_5_-OAc_3_	3180.15	3179.10	3179.15	[M-H]^−^
[ECA]_5_-GlcNAc-OAc	3299.21	3297.76	3298.21	[M-H]^−^
[ECA]_5_-ManNAcA-OAc	3313.19	3311.68	3312.18	[M-H]^−^
[ECA]_5_-ManNAcA-OAc_2_	3355.20	3353.50	3354.20	[M-H]^−^
[ECA]_4_-GlcNAc-OAc_2_	2734.00	2732.67	2732.99	[M-H]^−^
[ECA]_4_-GlcNAc-OAc	2691.99	2690.60	2690.98	[M-H]^−^
**Fraction 26 (negative mode)**				
[ECA]_4_-ManNAcA-OAc_3_	2789.99	2788.69	2788.98	[M-H]^−^
[ECA]_4_-ManNAcA-OAc_3_	2789.99	2770.66	2770.97	[M-H_2_O-H]^−^
[ECA]_4_-ManNAcA-OAc_2_	2747.98	2746.67	2746.97	[M-H]^−^
[ECA]_4_-GlcNAc-OAc_2_	2734.00	2732.72	2732.99	[M-H]^−^
[ECA]_4_-ManNAcA-OAc	2705.97	2704.71	2704.96	[M-H]^−^
[ECA]_4_-GlcNAc-OAc	2691.99	2690.72	2690.98	[M-H]^−^
[ECA]_4_-ManNAcA	2663.96	2662.73	2662.95	[M-H]^−^
[ECA]_4_-GlcNAc	2649.98	2648.74	2648.97	[M-H]^−^
**Fraction 27 (negative mode)**				
[ECA]_4_-ManNAcA-OAc_2_	2747.98	2746.71	2746.97	[M-H]^−^
[ECA]_4_-GlcNAc-OAc_2_	2734.00	2732.73	2732.99	[M-H]^−^
[ECA]_4_-GlcNAc-OAc	2691.99	2690.74	2690.98	[M-H]^−^
[ECA]_4_-ManNAcA	2663.96	2662.75	2662.95	[M-H]^−^
[ECA]_4_-GlcNAc	2649.98	2648.75	2648.97	[M-H]^−^
[ECA]_4_-OAc_3_	2572.93	2571.72	2571.92	[M-H]^−^
[ECA]_4_-OAc_2_	2530.92	2529.71	2529.91	[M-H]^−^
[ECA]-Gal_3_-Glc_2_-Hep_3_-Kdo-P_3_-Etn	2514.69	2513.66	2513.68	[M-H]^−^
[ECA]_4_-OAc	2488.91	2487.65	2487.90	[M-H]^−^
[ECA]_4_-OAc	2470.90	2469.62	2469.89	[M-H_2_O-H]^−^
[ECA]_4_	2446.90	2445.61	2445.89	[M-H]^−^
**Fraction 28 (negative/positive modes)**				
[ECA]_4_-ManNAcA-OAc_2_	2747.98	2746.72	2746.97	[M-H]^−^
[ECA]_4_-GlcNAc-OAc_2_	2734.00	2732.75	2732.99	[M-H]^−^
[ECA]_4_-ManNAcA-OAc	2705.97	2704.82	2704.96	[M-H]^−^
[ECA]_4_-GlcNAc-OAc	2691.99	2690.78	2690.98	[M-H]^−^
[ECA]_4_-ManNAcA	2663.96	2662.77	2662.95	[M-H]^−^
[ECA]_4_-GlcNAc	2649.98	2648.82	2648.97	[M-H]^−^
[ECA]_4_-OAc_3_	2572.93	2571.74	2571.92	[M-H]^−^
[ECA]_4_-OAc_3_	2554.92	2553.78	2553.91	[M-H_2_O-H]^−^
[ECA]_4_-OAc_2_	2530.92	2529.71	2529.91	[M-H]^−^
[ECA]-Gal_3_-Glc_2_-Hep_3_-Kdo-P_3_-Etn	2514.69	2513.73	2513.68	[M-H]^−^
[ECA]_4_-OAc_2_	2512.91	2511.68	2511.90	[M-H_2_O-H]^−^
[ECA]_4_-OAc	2488.91	2487.67	2487.90	[M-H]^−^
[ECA]_4_-OAc	2470.90	2469.70	2469.89	[M-H_2_O-H]^−^
[ECA]_4_	2446.90	2445.62	2445.89	[M-H]^−^
[ECA]_4_-OAc	2488.91	830.67	830.64	[M+3H]^3+^
[ECA]_4_-OAc_2_	2530.92	838.64	838.65	[M-H_2_O+3H]^3+^
[ECA]_4_-OAc_2_	2530.92	844.67	844.65	[M+3H]^3+^
[ECA]-Gal_3_-Glc_2_-Hep_3_-Kdo-P_3_-Etn	2514.69	839.34	839.24	[M+3H]^3+^
[ECA]_4_-OAc_3_	2572.93	858.67	858.65	[M+3H]^3+^
[ECA]_4_-OAc_3_	2572.93	852.68	852.65	[M-H_2_O+3H]^3+^
[ECA]-Gal_3_-Glc_2_-Hep_3_-Kdo-P_4_-Etn	2594.65	865.98	865.89	[M+3H]^3+^
[ECA]_4_-GlcNAc-OAc	2691.99	892.40	892.34	[M-H_2_O+3H]^3+^
[ECA]_4_-ManNAcA-OAc_3_	2771.98	925.06	925.00	[M-H_2_O+3H]^3+^

**Table 5:** Interpretation of negative-ion mode MALDI-TOF mass spectra of fraction 5 isolated from *E. coli* R3 LOS preparation.

Oligosaccharide Structure	Calculated Monoisotopic Mass (Da)	Observed Ion (*m/z*)	Calculated Ion (*m/z*)	Interpretation of the Ion
[ECA]_4_-FucNAc-OAc_3_	2760.02	2759.94	2759.01	[M-H]^−^
[ECA]_4_-FucNAc-OAc_3_	2760.02	2741.93	2740.99	[M-H_2_O-H]^−^
[ECA]_4_-FucNAc-OAc_2_	2718.01	2717.90	2717.00	[M-H]^−^
Glc_3_-Gal-GlcNAc-Hep_3_-Kdo-P_3_	1905.45	1904.49	1904.44	[M-H]^−^
Glc_3_-Gal-GlcNAc-Hep_3_-Kdo-P_3_-Etn	1948.49	1947.51	1947.48	[M-H]^−^
Glc_3_-Gal-GlcNAc-Hep_3_-Kdo-P_3_-Etn	1948.49	1929.53	1929.47	[M-H_2_O-H]^−^
Glc_3_-Gal-GlcNAc_2_-Hep_3_-Kdo-P_1_	1948.60	1947.63	1947.59	[M-H]^−^
Glc_3_-Gal-GlcNAc_2_-Hep_3_-Kdo-P_1_	1948.60	1929.60	1929.57	[M-H_2_O-H]^−^
Glc_3_-Gal-GlcNAc_2_-Hep_3_-Kdo-P_2_	2028.66	2027.59	2027.55	[M-H]^−^
Glc_3_-Gal-GlcNAc_2_-Hep_3_-Kdo-P_2_	2028.66	2009.58	2009.54	[M-H_2_O-H]^−^
Glc_3_-Gal-GlcNAc_2_-Hep_3_-Kdo-P_2_-Etn	2071.60	2070.68	2070.60	[M-H]^−^
Glc_3_-Gal-GlcNAc_2_-Hep_3_-Kdo-P_2_-Etn	207.160	2052.62	2052.58	[M-H_2_O-H]^−^
Glc_3_-Gal-GlcNAc_2_-Hep_3_-Kdo-P_3_-Etn	2151.57	2150.59	2150.56	[M-H]^−^
Glc_3_-Gal-GlcNAc_2_-Hep_3_-Kdo-P_3_-Etn	2151.57	2132.57	2132.55	[M-H_2_O-H]^−^
Glc_3_-Gal-GlcNAc-Hep_3_-GlcN-Kdo-P_1_	1906.58	1887.63	1887.56	[M-H_2_O-H]^−^
Glc_3_-Gal-GlcNAc-Hep_3_-GlcN-Kdo-P_1_	1906.58	1905.61	1905.58	[M-H]^−^
Glc_3_-Gal-GlcNAc-Hep_3_-GlcN-Kdo-P_2_	1986.55	1967.57	1967.53	[M-H_2_O-H]^−^
Glc_3_-Gal-GlcNAc-Hep_3_-GlcN-Kdo-P_2_	1986.55	1985.56	1985.54	[M-H]^−^
Glc_3_-Gal-GlcNAc-Hep_3_-GlcN-Kdo-P_2_-Etn	2029.59	2010.64	2010.57	[M-H_2_O-H]^−^
Glc_3_-Gal-GlcNAc-Hep_3_-GlcN-Kdo-P_2_-Etn	2029.59	2028.63	2028.59	[M-H]^−^
Glc_3_-Gal-GlcNAc-Hep_3_-GlcN-Kdo-P_3_-Etn	2109.56	2108.59	2108.55	[M-H]^−^

Three detected core OS R3 glycoforms were colored in green, blue, and red.

## Abbreviations

ECA	Enterobacterial common antigen
ECA_PG_	Phosphatidylglycerol-linked ECA
ECA_CYC_	Cyclic ECA
ECA_LPS_	Lipopolysaccharide-associated ECA
LPS	Lipopolysaccharide
LOS	Lipooligosaccharide
O-PS	O-specific polysaccharide
OS	Oligosaccharide
P	Phosphate group
PP	Pyrophosphate group
PPEtn	Pyrophosphorylethanolamine
OAc	O-acetyl group
ESI-IT MS	Electrospray ionization-ion trap mass spectrometry
MALDI-TOF MS	Matrix-assisted laser-desorption/ionization-time of flight mass spectrometry
NMR	Nuclear magnetic resonance
MS	Mass spectrometry
